# Improvement of chemotherapy induced colitis with serum-derived bovine immunoglobulin

**DOI:** 10.1186/1753-6561-6-S3-P26

**Published:** 2012-06-01

**Authors:** Gerald L Klein, Eric M Weaver

**Affiliations:** 1 Thomas J Long School of Pharmacy and Health Science at the University of the Pacific, USA; 2 Entera Health, Inc., Cary, NC, 27511, USA

## Background

Chemotherapy-induced diarrhea is a common problem, with pain, ulceration, bloating, vomiting and diarrhea occurring in about 40% of patients on a standard regimen and dose [1]. The pathophysiology of this problem is quite complex, involving multiple pathways including increases in pro-inflammatory mediators tumor necrosis factor- alpha (TNF-α) and interleukin-6 (lL-6) [2]. Serum-derived bovine immunoglobulin (SBI) has been used for years; first as a valued feed ingredient for weaned pigs where it has been shown to decrease mortality and morbidity and increase growth rates [3]; and then as a safe dietary supplement in humans [4]. In experimental animal models, SBI has been found to decrease both TNF -α and lL-6 in intestinal inflammation, and decrease intestinal permeability [5].

## Case history

This patient is a white female, 54 years of age, who developed a renal cell carcinoma and an unrelated adenocarcinoma of the ovary. She underwent a nephrectomy, empiric lymph node dissection, omentectomy, and bilateral salpingo-oophrectomy. A 6-week course of carboplatin and paclitaxel was prescribed for the treatment of the carcinoma of the ovary. Consistent with previous reports, this treatment regimen induced abdominal pain, nausea, vomiting, and diarrhea; resulting in a 12 kilogram weight loss. With the physician’s approval, she began using 2 g of SBI b.i.d as a food supplement.

## Results

After using SBI, this patient’s symptoms improved while she continued with chemotherapy. She gained 2.5 kilograms in the next 2 weeks, without side effects from SBI and was able to successfully complete her course of chemotherapy. SBI has previously been shown to decrease diarrhea and, recently, has been shown to decrease intestinal permeability [6].

## Conclusions

SBI may have improved this patient’s gastrointestinal complaints and maintenance of body weight. Clinical and nonclinical studies are warranted to determine the efficacy of this food product in decreasing the side effects associated with chemotherapy treatment.
